# Isolation Hospitals in Guernsey

**Published:** 1907-01-19

**Authors:** 


### ISOLATION HOSPITALS IN GUERNSEY.

It is not often that nurses find themselves in occupation of a Government fort, but such is the case in Guernsey at the present time, where Mont Crevelt Battery is now in use as a temporary isolation hospital. This fort, situated on an eminence overlooking St. Sampson's Harbour, formerly mounted three big guns. It was built in the reign of George IV., and is surrounded by a moat (now dry), and has a drawbridge connecting it with the hill, but the raising gear has been dismantled.

The outside walls are high and of enormous thickness, and the gun embrasures have been filled in to ensure proper isolation. At one end of the fort is an old martello tower, which serves now as a sea-water reservoir for watering the roads. On one side of it are the kitchen and larder, on the other the nurses' sitting-room.

The bedrooms for the staff are in the main building, the big room of which, formerly the soldiers' quarters, provides ample and comfortable accommodation for twelve patients. Between this building and the wall of the fort, looking seaward, a level grass plot makes a fine playground.

A laundry is provided, but is not now in use. The old magazine, with its original doors, one of massive copper and the inner one of open woodwork, is the sanctum of the porter, a hearty and genial old salt who is well satisfied with his quarters and somewhat unusual work.

The thickness of the walls of the buildings makes them very dry, and the whole place has been well kept up by the authorities, so as to be ready for immediate occupation when required. The King Edward Sanatorium, the main infectious hospital of the island, is most beautifully situated 200 feet above the sea-level, and enjoys a fine view of the distant French coast, the Casquets, and the other islands of the Norman archipelago.

It is a fine modern group of buildings, opened in 1902, and built upon the block system. The wards are very lofty and airy, and have large verandahs round them; the walls are finished with Parian cement, and have rounded corners. Electric light, bells, and radiators for use when required are provided; there is a small telephonic exchange for use between the wards and the administration block, and through it a patient can communicate from any bed in the hospital with any subscriber or call-office in the island.

The patients can also listen to the services in St. Peter Port Church, three miles away, and special receivers are provided for the purpose, so that sounds in the wards are not heard by and do not disturb other listeners.

Such facilities are somewhat unusual, but in Guernsey both the sanatorium and the telephone system belong to the States, or Government of the Island.

The administrative block is large, well furnished, and comfortable, and in addition to the ordinary accommodation two big rooms are provided where the friends of a patient "dangerously ill" may pass the night, if their home is distant from the sanatorium. The laundry is well equipped with machinery, and worked by a six-horse power motor: it is provided with a pressure boiler to heat the water required by means of high pressure steam. The disinfecting plant is of the latest Manlove Alliott vacuum type, and a small refuse-destructor is kept always going. A mortuary and "viewing room," with a glass screen between, is near the disinfecting block. The water-tower contains tanks for filtered soft, as well as drinking, water, and both supplies are laid on throughout the buildings; the tanks are filled by a double-action pump driven by an electric motor. The drainage is dealt with by a system of septic tanks. The grounds are well laid out with trees and flowers, and a croquet lawn has been levelled and turfed at the expense of a kindly and grateful patient. In this favoured and sunny climate patients may often be seen enjoying the fresh air in their beds on the verandahs, while the inhabitants of the "adjacent islands of Great Britain and Ireland" are shivering with cold, or experiencing other climatic discomforts.

The accommodation of the hospital is ample for about 40 patients. The existence of this hospital is largely due to the efforts of the late Jurat Ozanne, C.S.I., a distinguished member of the Royal Court of the island, whose name will be always held in affectionate regard and respect by all connected with it, and the States of Guernsey may well be proud of the fact that they have built, equipped, and maintained an isolation hospital which is equal to any similar institution of its size in any part of the world.

One may also mention to their credit that, if not the first, they were one of the very first public authorities to supply antitoxin free for the treatment of patients suffering from diphtheria.

